# *Mycobacterium tuberculosis* Transmission from Human to Canine

**DOI:** 10.3201/eid1012.040094

**Published:** 2004-12

**Authors:** Paul C. Erwin, David A. Bemis, Dianne I. Mawby, Scott B. McCombs, Lorinda L. Sheeler, Inga M. Himelright, Sandy K. Halford, Lois Diem, Beverly Metchock, Timothy F. Jones, Melisse G. Schilling, Bruce V. Thomsen

**Affiliations:** * Tennessee Department of Health, Knoxville, Tennessee, USA;; † University of Tennessee, Knoxville, Tennessee, USA;; ‡ Centers for Disease Control and Prevention, Atlanta, Georgia, USA;; § Department of Agriculture, Ames, Iowa, USA

**Keywords:** letter, tuberculosis, canine disease, veterinary, restriction fragment length polymorphism

**To the Editor:** This report is the first known of a case of epidemiologically associated tuberculosis (TB) in a human and a canine caused by the same strain, confirmed by genotyping. In Tennessee, a 71-year-old woman with a 3-week history of a productive, nonbloody cough was evaluated. She lived alone, and standard epidemiologic investigation of family members and other close contacts showed no apparent TB exposure. A TB skin test 20 years earlier had been negative. Chest radiograph showed infiltrates and atelectasis in the upper lobe of the right lung. A TB skin test resulted in a 14-mm area of induration. Sputum stained positive for acid-fast bacilli (AFB) and was positive for *Mycobacterium tuberculosis* by DNA probe and culture. The organism was sensitive to standard antitubercular medications.

Treatment was initiated with isoniazid, rifampin, and pyrazinamide. After 14 days of daily, directly observed therapy, the patient complained of nausea, vomiting, and diarrhea. Treatment adjustments were made, and therapy was completed 11 months later with a complete recovery.

Six months after the patient's TB diagnosis, she took her 3-1/2-year-old male Yorkshire Terrier to a veterinary clinic with cough, weight loss, and vomiting of several months' duration. The dog lived indoors and had been a constant companion to the patient for 3 years. Because of the owner's diagnosis, TB was suspected. The dog's initial sputum sample was negative on AFB staining and *M. tuberculosis* nucleic acid amplification assay.

Eight days after discharge from a referral veterinary teaching hospital with a presumptive diagnosis of TB, the dog was euthanized because of urethral obstruction. Liver and tracheobronchial lymph node specimens collected at necropsy were positive by AFB stain and positive for *M. tuberculosis* complex by polymerase chain reaction. Cultures of liver, lung, and kidney specimens were positive for *M. tuberculosis.* The *M. tuberculosis* isolates from the dog and its owner had an indistinguishable 10-band pattern by IS*6110*-based restriction fragment length polymorphism genotyping (Figure) (*1*).

**Figure:**
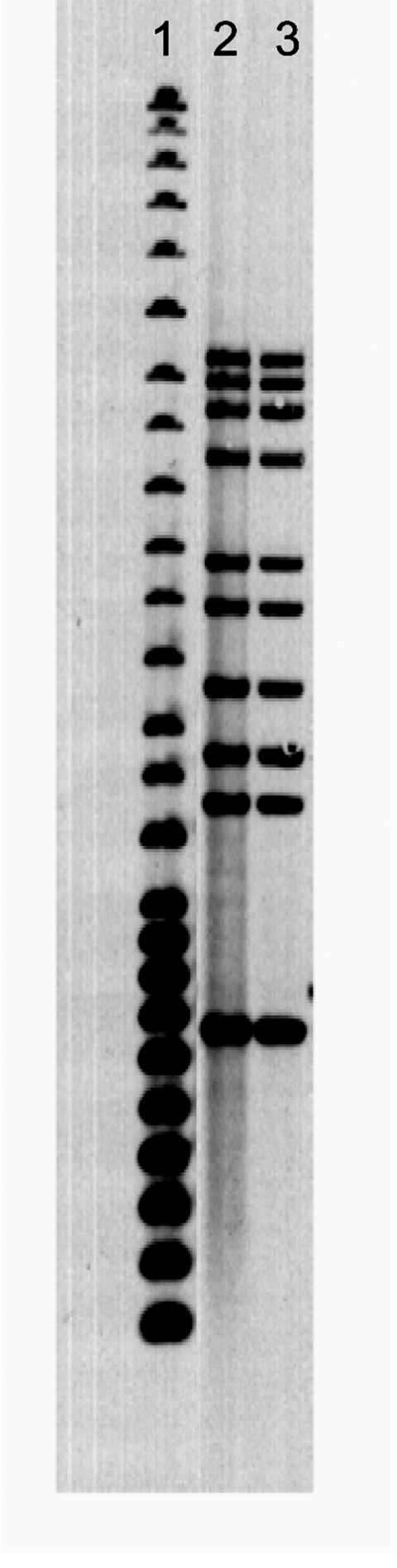
Ten-band *Mycobacterium tuberculosis* restriction fragment length polymorphism pattern. Lane 1, 25-band Centers for Disease Control and Prevention standard; lane 2, human case; lane 3, canine case.

This report is the first known of epidemiologically associated TB in human and canine cases to be confirmed by *M. tuberculosis* genotyping. The weight of historic data on human-canine TB supports our conclusion that the human owner was the likely source of the canine TB in this instance (*2*–*5*). In a review of 48 dogs and cats with known exposure to human TB, 7 (14.6%) were culture-positive for *M. tuberculosis* (*2*). Another series of eight canine TB infections documented by necropsy showed that seven of the dogs had a close association with humans with active TB disease (*5*). Other forms of mycobacterial infections, most notably *M. bovis*, have also been epidemiologically linked in humans and dogs (*4*,*6*). Cases in which dogs and cats infected with *M. bovis* or *M. tuberculosis* have infected humans have also been reported (*4*).

Genotyping has become a powerful tool for confirming epidemiologically linked transmission of *M. tuberculosis*. Two previous reports showed genotype matches between human and elephant TB cases and between human and monkey TB cases (*7*,*8*).

In our case, signs and symptoms that likely represented onset of TB appeared first in the woman and followed several weeks to months later in her pet dog. The owner often kept the dog in her lap, and the dog was allowed to lick the owner's face. A thorough, standard epidemiologic investigation did not identify any other infected contacts of the dog or owner; however, because the patient had limited social contacts, nonstandard investigation such as social network analysis was not formally conducted.

Cross-contamination of specimens was unlikely to have occurred at the laboratory at which cultures for *M. tuberculosis* were confirmed; several months passed between the times the two isolates were identified and subsequently sent to the Centers for Disease Control and Prevention for genotyping. This pattern has never been identified in a national database of >10,000 unique patterns, so this match is not likely to be due to anything other than transmission between the dog and its owner. Because systematic genotyping is not performed routinely in Tennessee, we are unable to determine more definitively whether this pattern has ever occurred in the state.

Although the true risk for TB transmission from humans to dogs, and vice versa, is not known, pet owners, physicians, and veterinarians should be aware of this potential. While standard tests, such as culture for *M. tuberculosis*, may be helpful in understanding the dynamics of TB between humans and other animals, genotyping has become the standard for confirming the association.
